# The introduction of regular group reflective practice sessions for junior doctors in a critical care setting during the second wave of COVID-19 pandemic – a Pilot

**DOI:** 10.1192/bjo.2021.359

**Published:** 2021-06-18

**Authors:** Itunuayo V Ayeni, Elizabeth Headon

**Affiliations:** 1 Oxleas NHS Foundation Trust, London; 2 Lewisham and Greenwich NHS Foundation Trust

## Abstract

**Aims:**

Reflective practice is a core component of undergraduate as well as post graduate training. Reflective practice provides an opportunity for individuals to learn through their experience as well as gaining insight into themselves and their practice. If completed effectively, it has been shown to reduce stress and improve mental well-being. Our aim therefore was to provide regular group reflective practice sessions with the aim of supporting junior doctor's mental wellbeing during the second wave of the COVID-19 pandemic.

**Method:**

Junior doctors within a critical care setting were offered two-weekly group reflective practice sessions focusing on ‘difficult or challenging cases and encounters.’ The sessions were offered to all junior and middle grade doctors within a critical care department in a small district general hospital. Consultants were also able to attend. The groups were facilitated by a consultant liaison psychiatrist and an accredited balint group leader. Critical care doctors were provided a feedback questionnaire assessing the impact of the sessions and the levels of stress and burnout. The themes emerging from the sessions were also explored.

**Result:**

A total of six reflective practice sessions were offered during a three-month period. A total of four reflective practice sessions were completed; two sessions were cancelled due to high workload on the department. Each session lasted approximately 50mins. On average a total of 3-4 junior doctors attended each session. The sessions were conducted face to face in a socially distanced manner and with all participants wearing face masks. The sessions were predominately attended by foundation doctors and SHOs. There was occasional attendance by middle grades and a consultant.

The predominant themes that emerged included: guilt, prolonged suffering, desensitisation, support and exhaustion. Despite the challenges associated with the pandemic and lockdown, many of the doctors also acknowledged the benefit of being at work during both waves of the pandemic. There was a sense of collectiveness and group belonging. The group found it beneficial to be able to share their experiences and challenges faced; this was most striking amongst the very junior members of the team.

Questionnaires were also provided to gain additional insight into the wellbeing of the critical care doctors. Worryingly the results highlighted a significant proportion of doctors were experiencing signs of burnout including fatigue (77%), lack of energy (54%), cynicism (31%), frustration and irritability (45%) and detachment (38%). Many of the issues highlighted were in response to the demand created by the pandemic and a lack of medical staffing wth 69% of doctors requesting regular feedback on staffing issues.

**Conclusion:**

Burnout and low morale were already highlighted in a significant number of junior doctors prior to the pandemic. COVID-19 has identified a clear need for NHS employers and medical leaders to provide emotional and psychological support to staff. It is vital that we create an open environment where individuals can express their feelings openly without fear that they will be judged. Group reflective practice provides an avenue to build on collectiveness created during both waves of the COVID-19 pandemic. This pilot has demonstrated that if introduced as part of a wellbeing support package, junior doctors within a critical care setting are able to utilise the sessions in an effective and productive manner.

